# Cloning, expression and identification of KTX-Sp4, a selective Kv1.3 peptidic blocker from *Scorpiops pococki*

**DOI:** 10.1186/s13578-017-0187-x

**Published:** 2017-11-06

**Authors:** Yan Zou, Feng Zhang, Yaxian Li, Yuanfang Wang, Yi Li, Zhengtao Long, Shujuan Shi, Li Shuai, Jiukai Liu, Zhiyong Di, Shijin Yin

**Affiliations:** 1 School of Pharmaceutical Sciences, South-Central University for Nationalities, Wuhan, 430074 People’s Republic of China; 2 School of Life Sciences, University of Science and Technology of China, Hefei, 230027 People’s Republic of China; 3 National Demonstration Center for Experimental Ethnopharmacology Education, South-Central University for Nationalities, Wuhan, 430074 People’s Republic of China

**Keywords:** Peptide KTX-Sp4, *Scorpiops pococki*, Kv1.3, Channel turret, Selectivity

## Abstract

**Background:**

Specific and selective peptidic blockers of Kv1.3 channels can serve as a valuable drug lead for treating T cell-mediated autoimmune diseases, and scorpion venom is an important source of kv1.3 channel inhibitors. Through conducting transcriptomic sequencing for the venom gland of *Scorpiops pococki* from Xizang province of China, this research aims to discover a novel functional gene encoding peptidic blocker of Kv1.3, and identify its function.

**Results:**

We screened out a new peptide toxin KTX-Sp4 which had 43 amino acids including six cysteine residues. Electrophysiological experiments indicated that recombinant expression products of KTX-Sp4 blocked both endogenous and exogenous Kv1.3 channel concentration-dependently, and exhibited good selectivity on Kv1.3 over Kv1.1, Kv1.2, respectively. Mutation experiments showed that the Kv1 turret region was responsible for the selectivity of KTX-Sp4 peptide on Kv1.3 over Kv1.1.

**Conclusions:**

This work not only provided a novel lead compound for the development of anti autoimmune disease drugs, but also enriched the molecular basis for the interaction between scorpion toxins and potassium channels, serving as an important theoretical basis for designing high selective Kv1.3 peptide inhibitors.

## Background

About 7% of the population are seriously threatened by nearly 80 kinds of autoimmune diseases such as rheumatoid arthritis, systemic lupus erythematosus and type 1 diabetes [1]. As traditional immunosuppressants, steroids [2] and cyclophosphamides [3] have been widely used to treat autoimmune diseases. However, they often cause side effects, such as reducing the patient’s normal protective immune response and increasing the risk of infection. Therefore, inhibiting the abnormal autoimmune reaction and maintaining the normal protective immune response is a big challenge in the treatment of autoimmune diseases [4]. More and more researches have demonstrated that the pathogenesis of autoimmune diseases involves activation and proliferation of effector memory T cells (T_EM_ cells) [5]. During the activation of T_EM_ cells, the expression of the Kv1.3 channel was up-regulated significantly, from about 300 molecules to about 1500–2000 molecules per cell [6]. Selective blockage of Kv1.3 channels was experimentally demonstrated to suppress T_EM_ cell proliferation [7]. There is also a growing body of evidence suggesting that Kv1.3 channel blockers have beneficial therapeutic effect on rheumatoid arthritis [8], autoimmune encephalitis [9] and other autoimmune diseases [10]. With the establishment of Kv1.3 channel as an excellent drug target for autoimmune diseases, extensive efforts have been made to develop selective and efficient Kv1.3 channel blockers and provide lead drugs for the treatment of autoimmune diseases.

Toxin peptides from natural venomous animals comprise the largest families of ion channel blockers, and they are becoming increasingly valuable sources of new drugs for channelopathies. Scorpion is one of the oldest species that have existed on earth for more than 400 million years. A large number of studies have showed that scorpion venom contains many short peptides with 20-80 amino acid residues, which is an important source of kv1.3 channel inhibitors [11]. For scorpion species which can be farmed on a large scale, such as *Buthus* *martensii* Karsch, high abundance active polypeptides can be directly separated and extracted from scorpion venom. However, for low abundance scorpion toxin polypeptide or for scorpion species which cannot be cultured in large scale, it is difficult to extract the active polypeptide directly from scorpion venom. Since transcriptomic technique has been proved to be one of the most powerful strategies for screening functional genes from the venom glands of scorpions [12, 13], the combination of modern transcriptome sequencing and genetic engineering techniques can effectively overcome this difficulty. In this study, we screened a scorpion toxin *KTX*-*Sp4* gene by transcriptome sequencing from the venom glands of *Scorpiops pococki* from Xizang province. The peptides coded by *KTX*-*Sp4* gene have a high homology with Kv1.3 channel inhibitors HLKTx4 [14], J123 [15], pMeKTx22-1 and LmKTx8 [16]. Whole cell patch-clamp experiments indicated that peptide KTX-Sp4 had potentially selective blocking effect on Kv1.3 over Kv1.1 channel, and the selective recognition of KTX-Sp4 on Kv1.3 over Kv1.1 was determined by four different amino acid residues in the turret region between Kv1.1 and Kv1.3 channels.

## Methods

### Transcriptome sequencing and data analysis

*Scorpiops* *pococki* were collected in the XiZang Province of China and identified by Dr. Zhiyong Di (University of Science and Technology of China). Glands of *Scorpiops pococki* were collected 2 days after electrical extraction of their venom. Total RNA was prepared from 5 glands, using Trizol reagent (Invitrogen) method. The RNA samples were subsequently treated with RNase-Free DNase I (Qiagen, USA) to eliminate genomic DNA. Finally, high-quality RNA samples (RNA concentration ≥ 1200 ng/μl, RNA Integrity Number ≥ 9.0) were used for further construction of cDNA libraries. The cDNA libraries of *Scorpiops pococki* were sequenced using Illumina HiSeqTM 2000 platform (San Diego, CA, USA) by BGI-Shenzhen. BLASTx or BLASTn alignment (e-value 10^−5^) was performed to search achieved unigenes of *Scorpiops pococki* from six public databases, including Non-redundant (Nr), Swiss-prot protein (Swiss-Prot), Kyoto Encyclopedia of Gene and Genomes (KEGG), Cluster of Orthologous Group of proteins (COG) and Non-redundant nucleotide database (Nt). For prediction of unigene functions, we used Blast2GO program to annotate unigenes and obtain corresponding Gene Ontology Consortium (GO) annotation for each unigene.

### Construction of expression vector pGEX-4T-1-KTX-Sp4

**Fig. 1:**
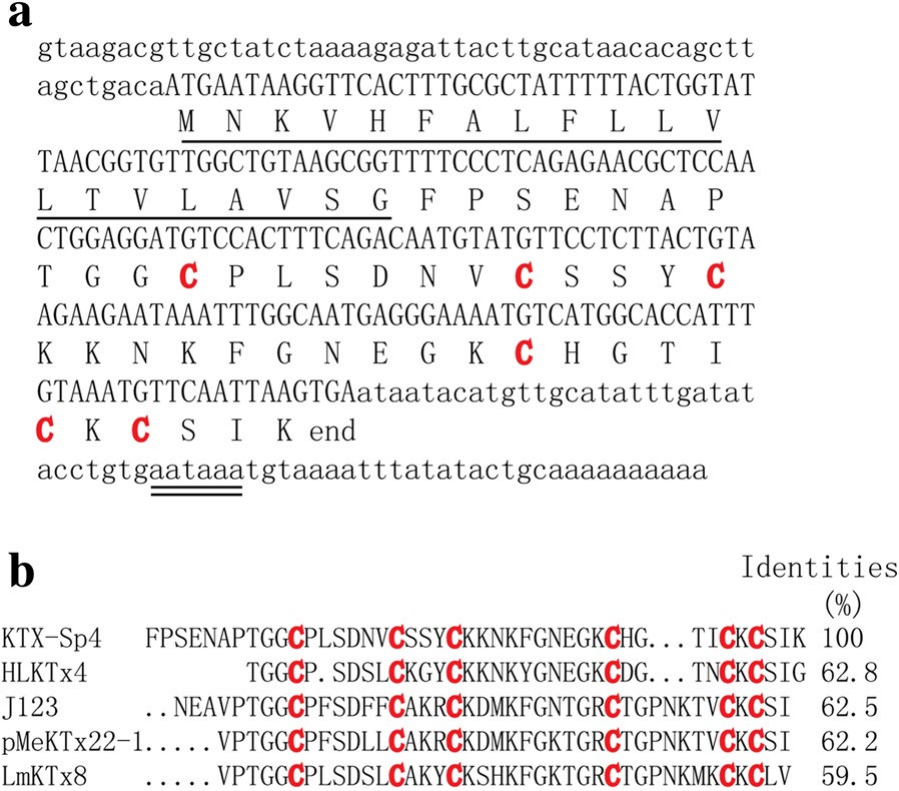
**a** Full-length nucleotide sequences and the corresponding amino acids of KTX-Sp4. The signal peptide is underlined, while the potential polyadenylation signal AATAAA is underlined twice. Red colors indicate the cysteine residues, 5′ and 3′ UTR regions are in lowercase letters. The numbers to the right mean the order of amino acids. **b** Sequence alignments of peptide KTX-Sp4 with the nearest neighbors

Expression plasmid pGEX-4T-1-KTX-Sp4 was constructed on the basis of the full-length cDNA of KTX-Sp4 (Fig. 1), a predicted functional gene from the GO annotation of *Scorpiops pococki*. Primers were designed to match the mature region of KTX-Sp4. A second PCR used the products of the overlapping PCR as templates. The primers used were: Sense primer 1,5′-CTGGGATCC GATGACGATGACAAGGCGTTTCCGAGCGAAAACCCGACCGGCG-3′ with a *Bam*HI restriction enzyme site (underlined) and corresponding to five codons encoding an enterokinase cleavage site (underlined twice); Sense primer 2,5′-AAAACCCGACCGGCGGCTGCCCGCTGAGCGATAACGTGTGCAGCAGCTATTG-3′; Antisense primer 1,5′-CCGCTCGAGTCATTTAATGCTGCATTTGCAAATGGTGCCATGGCATTTGCCTTC-3′; Antisense primer 2, 5′-TATGGCATTTGCCTTCGTTGCCAAATTTGTTTTTTTTGCAATAGCTGCTGCAC-3′ with *Xho*I restriction enzyme site (underlined). The PCR products were inserted into expression vector pGEX-4T-1. The plasmid were sequenced with universal pGEX primers. *E. coli* Rosetta (DE3) cells were used for expression.

### Expression and purification of KTX-Sp4 peptides

*Escherichia coli* Rosetta (DE3) cells containing pGEX-4T-1-KTX-Sp4 were proliferated at 37 °C in LB with 100 mg/ml ampicillin. Fusion protein synthesis was induced by the addition of 0.5 mM isopropyl β-D-thiogalactoside (IPTG) at 28 °C for 4 h. Cells were harvested and resuspended in glutathione (GSH) wash buffer (pH 8.0, 50 mM Tris–HCl, 10 mM EDTA), digested by 1 mg/ml lysozyme for 30 min. After a brief sonication, the extract was clarified by a centrifugation at 10,000×*g* for 15 min. The fusion protein was purified by GSH affinity chromatography and enriched by centrifugal filter devices (Millipore, 10 kDa). High performance liquid chromatography (HPLC) was used to further purify peptide, under the 230 nm wavelength to monitor the absorbance of the eluate at room temperature (22–25 °C). After cleavage of the fusion protein by enterokinase (More Biotechnology, Wuhan) for 8 h at 37 °C, the mixture was filtered (Millex-HV, 0.45 mm, Millipore) and separated on a C18 column (EliteHPLC, China, 10 mm × 250 mm, 5 μm) using a linear gradient from 10 to 80% CH_3_CN with 0.1% TFA in 60 min with a constant flow rate of 5 ml/min. Peaks were collected manually.

### Cell isolation, culture and potassium channels expression

Mouse spinal columns were removed and placed in ice-cold HBSS; neurons were acutely dissociated and maintained as described [17]. In brief, laminectomies were performed and bilateral DRG were dissected out. After removal of connective tissues, DRG were transferred to a 1 ml Ca^2+^/Mg^2+^-free HBSS containing 2 μl saturated NaHCO_3_, 0.35 mg l-cysteine and 20 U papain (Worthington, Lakewood, NJ, USA), and incubated at 37 °C for 10 min. The suspension of DRG was centrifuged, the supernatant was removed, 1 ml Ca^2+^/Mg^2+^-free HBSS containing 4 mg collagenase type II and 1.25 mg dispase type II (Worthington) was added and incubated at 37 °C for 10 min. After digestion, neurons were pelleted, suspended in neurobasal medium containing 2% B-27 supplement, 1% l-glutamine, 100 U/ml penicillin plus 100 μg/ml streptomycin, and 50 ng/ml nerve growth factor, plated on a 12 mm coverslip coated with poly-l-lysine (10 μg/ml) and cultured under a humidified atmosphere of 5% CO_2_/95% air at 37 °C for 18–24 h before use.

Jurkat E6-1 T cells (ATCC TIB152) and HEK293T cells (ATCC ACS4500) were maintained in RPMI medium 1640 (Invitrogen, Carlsbad, CA, USA) and Dulbecco’s modified Eagle’s medium (DMEM) (Life Technologies, GrandIsland, NY, USA), supplemented with 10% fetal bovine serum (Life Technologies), 100 units/ml penicillin, 100 μg/ml streptomycin, respectively. Cells were cultured in a humidified incubator at 37 °C with 5% CO_2_. The cDNAs encoding mKv1.1, mKv1.1-AEHS/PSGN, hKv1.2 and mKv1.3 [18] were subcloned into the *Xho*I/*Bam*HI sites of a bicistronic vector, pIRES_2_-EGFP (Clontech, USA), then transiently transfected into HEK293-T cells using Lipofectamine 2000 (Invitrogen) for electrophysiological experiments.

### Electrophysiological recordings and data analysis

Whole-cell patch-clamp recordings were performed using an EPC 9 amplifier (HEKA Elektronik, Lambrecht/Pfalz, Germany) at room temperature (22–24 °C). Pipettes pulled from borosilicate glass (BF 150-86-10; Sutter Instrument Company, Novato, CA, USA) had resistances of 2–4 MΩ when filled with the internal solution. The internal pipette solution for recording Kv currents on DRG neurons contained: 120 mM potassium gluconate, 20 mM KCl, 10 mM EGTA, 10 mM HEPES, 5 mM Na_2_-ATP, 2 mM MgCl_2_, 1 mM CaCl_2_ (pH 7.3 withKOH). The external solution for recording Kv currents on DRG neurons contained: 150 mM choline chloride, 10 mM HEPES, 10 mM glucose, 5 mM KCl, 2 mM CaCl_2_, 2 mM MgCl_2_, 1 mM CdCl_2_ (pH 7.4 withKOH). The other internal pipette and external solutions were prepared according to the previous procedures [19]. Kv currents were elicited by + 50 mV, 400 ms depolarizing pulse from the holding potential of -60 mV every 20 s. Using IGOR (WaveMetrics, Lake Oswego, OR) software, concentration–response relationships were fitted according to modified Hill equation: *I*_toxin_/*I*_control_ = 1/1 + ([peptide]/IC_50_), where *I* is the steady-state current and [peptide] is the concentration of toxin. The parameter to be fitted was concentration of half-maximal effect (IC_50_).

## Results

### Sequence analysis of KTX-Sp4

By conducting transcriptome sequencing for *Scorpiops pococki* venom glands, one of the nucleotide sequences obtained displayed an ORF encoding a new putative neurotoxin which was termed *KTX*-*Sp4*. The precursor nucleotide sequence of *KTX*-*Sp4* is 312 bp in length, including three parts: 5′UTR, ORF and 3′UTR. The 5′ and 3′ UTRs of KTX-Sp4 are 53 and 67 bp in length (Fig. 1a), respectively. At the 3′UTR end of the cDNA, a single AATAAA polyadenylation signal is found 19 nt upstream of the poly(A) tail. An ORF which is 192 bp in length encodes a precursor of 63 amino acid residues (Fig. 1a). SignalP V3.0 server (http://www.cbs.dtu.dk/services/SignalP/) predicted that the precursor of KTX-Sp4 contained a putative signal peptide of 20 residues following a mature toxin of 43 residues with three pairs of disulfide bridges. By sequence alignment with the other toxins (Fig. 1b), it is reasonable to assume that KTX-Sp4 adopts the well-known cysteine-stabilized α/β scaffold, which is similar to the scorpion classical K^+^-channel blockers. The KTX-Sp4 was found identical with HLKTx4 [14], J123 [15], pMeKTx22-1 and LmKTx8 [16] by 62.8, 62.5, 62.2 and 59.5%, respectively. KTX-Sp4 may have similar function with blocking Kv1.3 channels, yet it is necessary to investigate the biological effect of KTX-Sp4 peptide by electrophysiological experiments for identifying its specific target.

### Expression, purification and characterization of KTX-Sp4 peptide

**Fig. 2:**
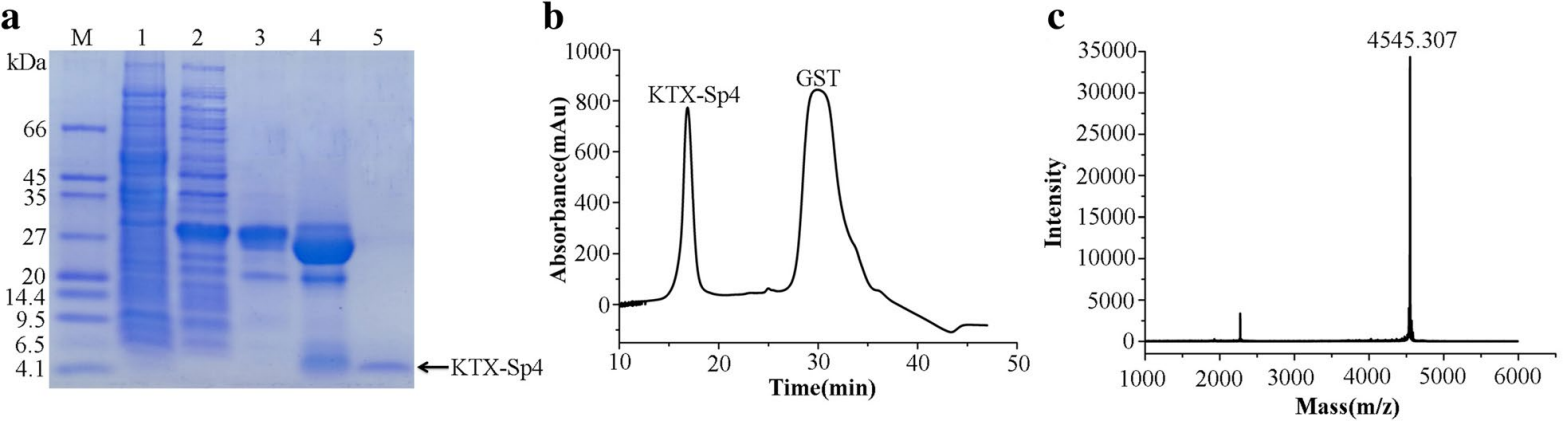
The expression, purification and identification of peptide KTX-Sp4. **a** Tricine/SDS-PAGE analysis of the purification of KTX-Sp4 peptide. M, molecular mass markers; Lane 1, proteins from non-induced coli Rosetta (DE3) cells; lane 2, proteins from induced coli Rosetta (DE3) cell containing pGEX-4T-1-KTX-Sp4 by IPTG; lane 3, purified GST fusion protein after affinity chromatography and desalting; lane 4, fusion protein cleaved by enterokinase; lane 5, purified KTX-Sp4 by reversed phase HPLC. **b** Purification of KTX-Sp4 by HPLC on a C18 column. **c** Mass spectrum of KTX-Sp4 peptide measured by MALDI-TOF–MS. Measured value is 4545.3 Da, and the calculated one is 4547.3 Da

The expressed GST-KTX-Sp4 fusion protein was purified on GSH affinity column and then desalted using centrifugal filter devices. The fusion protein was cleaved into GST protein and KTX-Sp4 peptides by enterokinase. As shown in Fig. 2a, the fusion protein of 31 kDa size was purified successfully and split into two products, the GST in 26 kDa and another protein in 4.5 kDa. The mixture was further separated by HPLC, resulting in two peaks (Fig. 2b). The component eluting at about 16 min and corresponding to KTX-Sp4 was collected manually and lyophilized. The molecular weight of KTX-Sp4 was determined by matrix assisted-laser-desorption/ionization time-of-flight mass spectrometry (MALDI-TOF–MS). Results showed that the measured value of KTX-Sp4was 4545.3 Da (Fig. 2c), which confirmed the theoretical molecular weight of 4547.3 Da.

### Modulation of KTX-Sp4 on endogenous voltage-gated potassium channels

Primary structure sequence alignments showed that KTX-Sp4 polypeptide had high homology with J123 and LmKTx8 (Fig. 1), which suggested that KTX-Sp4 may also have the function of blocking Kv1.3 channels. We first examined whether KTX-Sp4 could block endogenous Kv1.3 expressed by human Jurkat T cells. To avoid activation of the SKCa2 channel, a pipette solution containing almost zero cytosolic Ca^2+^ was adopted. Kv1.3-mediated currents were elicited by 400 ms depolarizing pulses from a holding potential of − 60 to + 50 mV. Bath application of KTX-Sp4 reduced Kv1.3 currents by concentration-dependence with the IC_50_ of 235.02 ± 3.36 nM. The suppressive effect of KTX-Sp4 on Kv1.3 was partially reversible after washout.

**Fig. 3:**
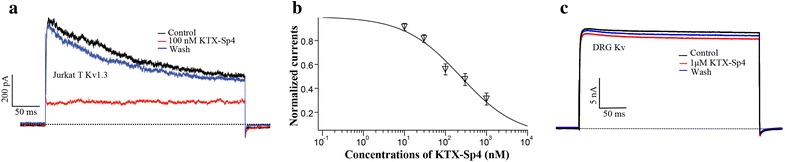
Modulation of KTX-Sp4 on endogenous voltage-gated potassium channels. **a** Representative traces illustrate that 100 nM KTX-Sp4 inhibited the Kv1.3 current in a Jurkat T cell reversibly. **b** Concentration–response curve of KTX-Sp4 inhibition of Kv1.3 current in Jurkat T cells. Currents were normalized to the control and fitted by a Hill equation; IC_50_ value was 235.02 ± 3.36 nM (n = 8). **c** Current traces of voltage-gated potassium channels in DRG cells in the absence (control) or presence of 1 μM KTX-Sp4

Since there are no expression or weak expression of Kv1.3 channels on DRG cell membranes [20], this study will detect whether KTX-Sp4 could modulate the other voltage-gated potassium channel which is expressed on DRG cell membranes. On acutely isolated DRG cells, voltage-gated potassium channels currents were elicited by 400 ms depolarizing pulses from a holding potential of − 60 to + 50 mV. As showed in Fig. 3, even at the high concentrations of 1 μM, KTX-Sp4 has no significant inhibitory effect on voltage-gated K^+^ currents on DRG cells. Above results preliminary prove that KTX-Sp4 can block the endogenous Kv1.3 channels selectively on Jurkat T cells.

### Selective blocking of KTX-Sp4 on exogenous Kv1.3 channel

We also investigated the inhibitory effect of KTX-Sp4 on Kv1.3 channels heterologously expressed in HEK293T cells. As expected, KTX-Sp4 reduced the peak amplitude of wild-type mKv1.3-mediated currents in a concentration-dependent manner, which reappeared the phenomenon in the Jurkat T cells. The steady-state current measured at the end of the depolarizing pulse was markedly decreased by KTX-Sp4 with an IC_50_ of 24.73 ± 10.8 nM (n = 10).

Mammalian Kv1.1 and Kv1.2 are highly homologous to the Kv1.3 channel, which affects the selectivity of Kv1.3 channel blockers. Therefore, we also observed the effect of KTX-Sp4 peptides on Kv1.1 and Kv1.2 channels heterologously expressed in HEK293T cells. The addition of 1 μM KTX-Sp4 only reduced the maximum currents of Kv1.1 and Kv1.2 channel by about 20.85 and 7.23%, respectively, which indicated the good selectivity of KTX-Sp4 on Kv1.3 over Kv1.1 and Kv1.2. These electrophysiological results suggested that KTX-Sp4 could serve as a potential drug lead for selectively targeting Kv1.3 channel, thus playing a beneficial role in drug design for treating autoimmune diseases.

Among all mammalian ion channels, Kv1.1 has the highest homology to Kv1.3. The different residues in Kv1 turret region can well explain the selective blocking effect of some bioactive polypeptid on Kv1.3 over Kv1.1 [18]. To further elucidate the selective inhibition of KTX-Sp4 on Kv1.3 over Kv1.1, we constructed mutant Kv1.1-AEHS/PGSN by replacing four different amino acid residues in the Kv1.1 channel turret region with those in Kv1.3 channel. Compared to that on wild-type Kv1.1, the inhibitory effect of KTX-Sp4 on Kv1.1-AEHS/PGSN was significantly increased with an IC_50_ of 12.07 ± 4.08 nM, which is very close to the IC_50_ of wild Kv1.3 channel. Electrophysiological experiments showed that similar to ADWX-1 [18], the selective recognition of KTX-Sp4 on Kv1.3 over Kv1.1was also determined by the different residues in the turret region between Kv1.1 and Kv1.3 channels.

## Discussion

Venomous animals like scorpions [21], snakes [22], spiders [23] capture prey and defense themselves through secretion of venom containing ion channel regulatory peptides. These peptides are not only excellent molecular probes for studying the physiological function and structure of ion channels, but also important resources for screening lead compounds of ion channel targeted drugs. As one of the most ancient species, scorpions have four hundred million years of evolutionary history. There are about 2000 species of scorpions in the world, and about 50-100 different toxin peptides are supposed to exist in each scorpion venom [12]. Since there are fewer kinds of scorpion species being used to discover bioactive peptides, only about 0.4–0.5% of toxin peptides have been identified and clearly characterized so far [24]. Till now, the reported toxin polypeptide extracted from Xizang *Scorpiops pococki* has been far less than that from other scorpions such as *Buthus martensii* Karsch [25, 26]. By combined use of transcriptome sequence and functional gene prediction in present work, the *KTX*-*Sp4* gene in the cDNA Library of Xizang *Scorpiops pococki* was screened, and then successfully expressed in prokaryotic expression system. Electrophysiological experiments showed that KTX-Sp4 peptides had a distinctively selective blocking effect on Kv1.3 channel—a new therapeutic target of autoimmune diseases.

**Fig. 4:**
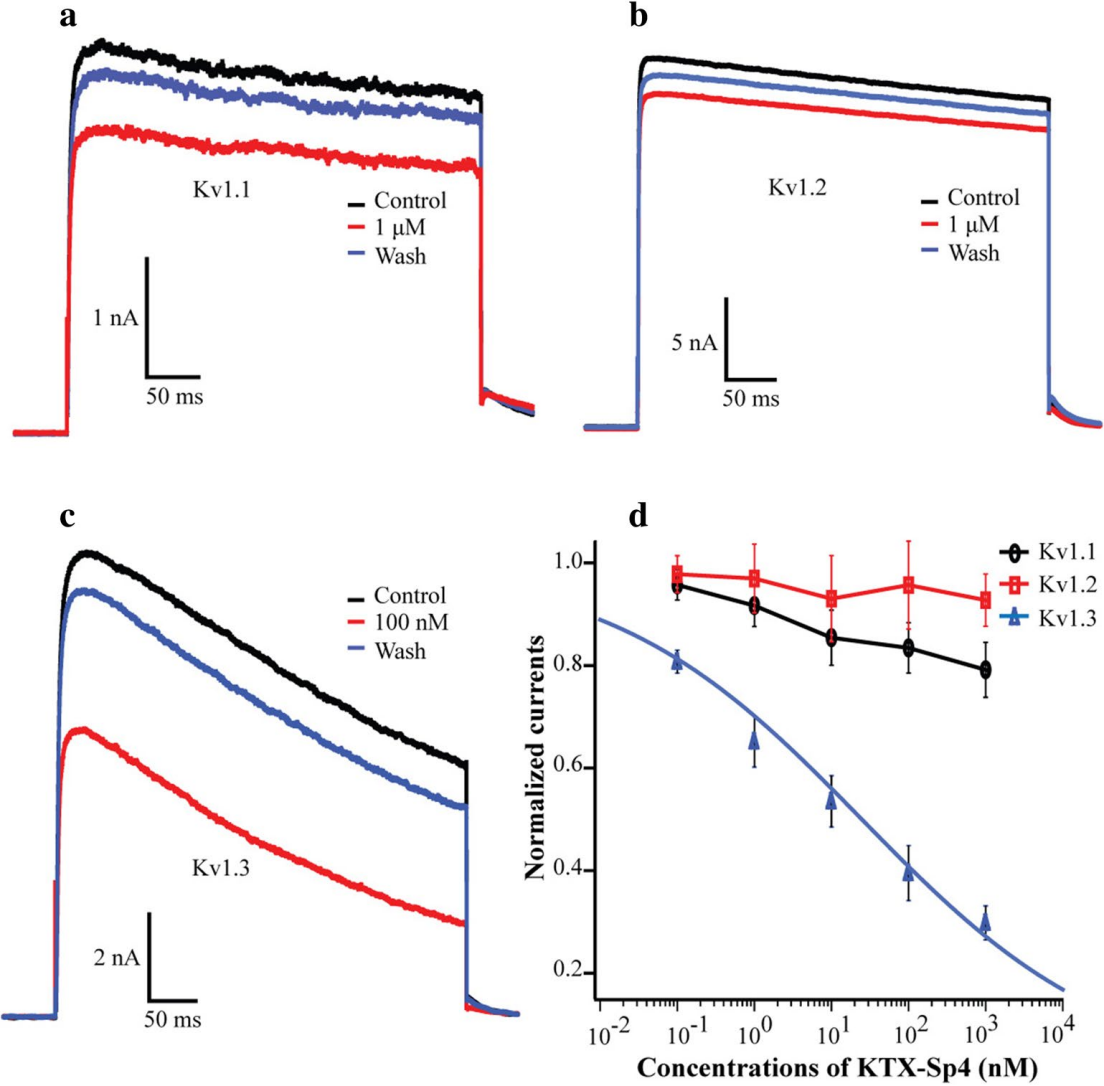
Inhibiting effect of peptide KTX-Sp4 on exogenous Kv1.x channels. **a**–**c** Current traces in the absence (control) or presence of KTX-Sp4 on Kv1.1, Kv1.2 and Kv1.3 channels: **a** 1 μM KTX-Sp4 on Kv1.1, **b** 1 μM KTX-Sp4 on Kv1.2, **c** 100 nM KTX-Sp4 on Kv1.3. **d** Average normalized current inhibition by various concentrations of KTX-Sp4 for Kv1.1, Kv1.2 and Kv1.3 channels, as indicated. Data represent mean ± SE of at least five experiments

KTX-Sp4 was found identical with J123 [15] and LmKTx8 [16] in primary structure by 62.5 and 59.5%, respectively. In addition, KTX-Sp4 can also block Kv1.3 channel functionally. This experiment phenomenon further demonstrated the principle that homologous proteins were similar in structure and function. The blocking activity of KTX-Sp4 on Kv1.3 was 31 times weaker than that of J123, while was comparable to that of LmKTx8. Scorpion toxin peptides used positively charged residues to identify and bind Kv1.3 S5-S6 pore link region where is rich in negatively charged residues, while used negatively charged residues to orient the recognization [27, 28]. Different distributions of positively and negatively charged residues between KTX-Sp4 and J123 might be the structural basis for explicating their functional differences on blocking Kv1.3 channel. As shown in Fig. 1, corresponding to the Tyr^21^ of KTX-Sp4, LmKTx8 has the same residue, while J123 has a positively charged residue Arg. In addition, corresponding to the Lys^24^ of KTX-Sp4, J123 has a negatively charged residue Asp. In selective blocking on Kv1.3 over Kv1.2, the KTX-Sp4 is superior to J123. As shown in Fig. 4, 1 μM KTX-Sp4 only has a weak blocking effect on Kv1.2, but J123 shows strong blocking activity on Kv1.2, with the IC_50_ of 26.4 ± 9.3 nM. As shown in Fig. 1, three residues Pro, Asn and Lys (PNK) existing in J123 and LmKTx8 are absent between Gly^35^ and Thr^36^ of KTX-Sp4, which suggests that these three PNK residues might be the important structure for J123 to recognize Kv1.2 channel, resulting in low selective blocking of J123 on Kv1.3 over Kv1.2. The spatial structure analysis and amino acid residues mutagenesis would help to thoroughly elucidate the different function between KTX-Sp4 and J123, and then contribute to the molecular design of highly selective and highly active Kv1.3 peptide blockers.

**Fig. 5:**
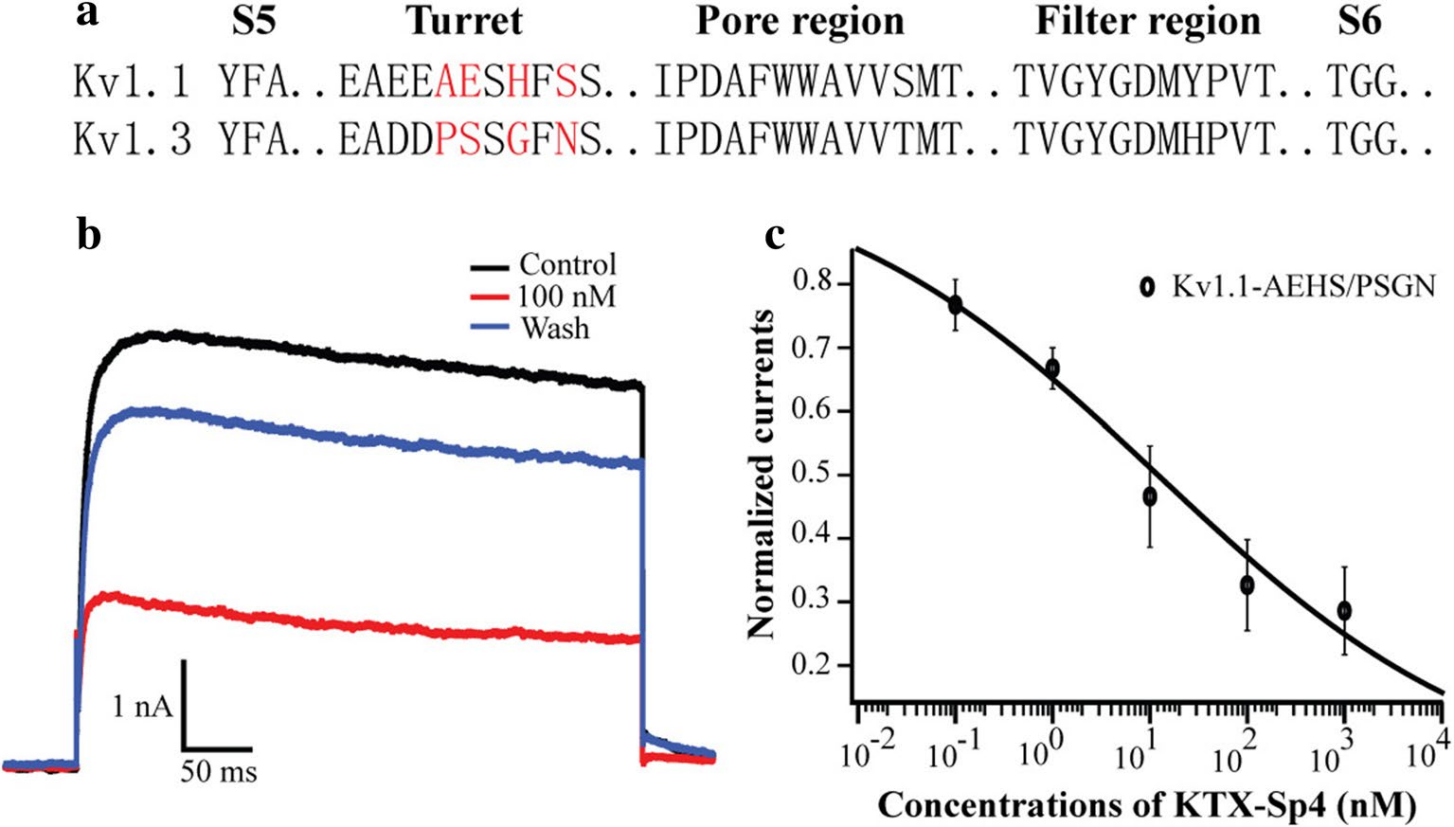
Affinity of KTX-Sp4 for the turret region mutant of Kv1.1. **a** Sequence alignments of amino acid residues in the S5-S6 link region between Kv1.1 and Kv1.3. Red letters indicate different amino acid residues in the turret region between Kv1.1 and Kv1.3. **b** Current traces in the absence (control) or presence of 100 nM KTX-Sp4 on Kv1.1-AEHS/PSGN channels. **c** Normalized current inhibition by various concentrations of KTX-Sp4 on Kv1.1-AEHS/PSGN channels. Data represent mean ± SE of six experiments

Among all mammalian ion channels, Kv1.1 is the most homologous channel with Kv1.3. As a result, the lack of selectivity for Kv1.1 and Kv1.3 channels restricts the further development and application of many Kv1.3 channel blockers [18]. Selectivity improvement of peptide drug lead between Kv1.1 and Kv1.3 remains a big challenge. Our group previously reported that the residues on the Kv1 turret region were responsible for the selectivity of ADWX-1 on Kv1.3 over Kv1.1 [18]. Since KTX-Sp4 displayed the significant selectivity on Kv1.3 over Kv1.1, did the different turret region also determine the selective regulation of KTX-Sp4 on Kv1.3 over Kv1.1? Turret region mutation experiments gave the positive and interesting answer. A mutant of the Kv1.1 channel (Kv1.1-AEHS/PSGN), in which four key residues of the Kv1.1 turret were replaced by the corresponding residues in Kv1.3 turret (Fig. 5), had a similar sensitivity to KTX-Sp4 as the Kv1.3. KTX-Sp4 and ADWX-1 only shared 16.28% homology, but the mechanism for selectively regulating on Kv1.3 over Kv1.1 had more common characteristics, which suggests that different turret regions between Kv1.3 and Kv1.1 should be considered in the molecular design of highly selective Kv1.3 channel peptide blockers.

## Conclusions

With selective inhibition on Kv1.3 channels, KTX-Sp4 peptide is a novel lead compound for the development of anti-autoimmune disease drugs. It can be known from this work that different structures between KTX-Sp4 and J123 led to different biological activities and Kv1turret region determined the selective regulation of KTX-Sp4 on Kv1.3 over Kv1.1, which enriches the molecular basis of the interaction between scorpion toxins and potassium channels, and also provides important theoretical basis for designing high selective Kv1.3 channel inhibitors.
